# Supplementary material for: Apical size reduction by macropinocytosis alleviates tissue crowding
Source: Nat Commun. 2025 Jun 23;16:5338. doi: 10.1038/s41467-025-60724-2 (PMC12185762; doi:10.1038/s41467-025-60724-2)
Supplement: Supplementary file 1 — Supplementary Information [file 41467_2025_60724_MOESM1_ESM.pdf]

APICAL SIZE REDUCTION BY MACROPINOCYTOSIS ALLEVIATES TISSUE CROWDING

SUPPLEMENTARY INFORMATION

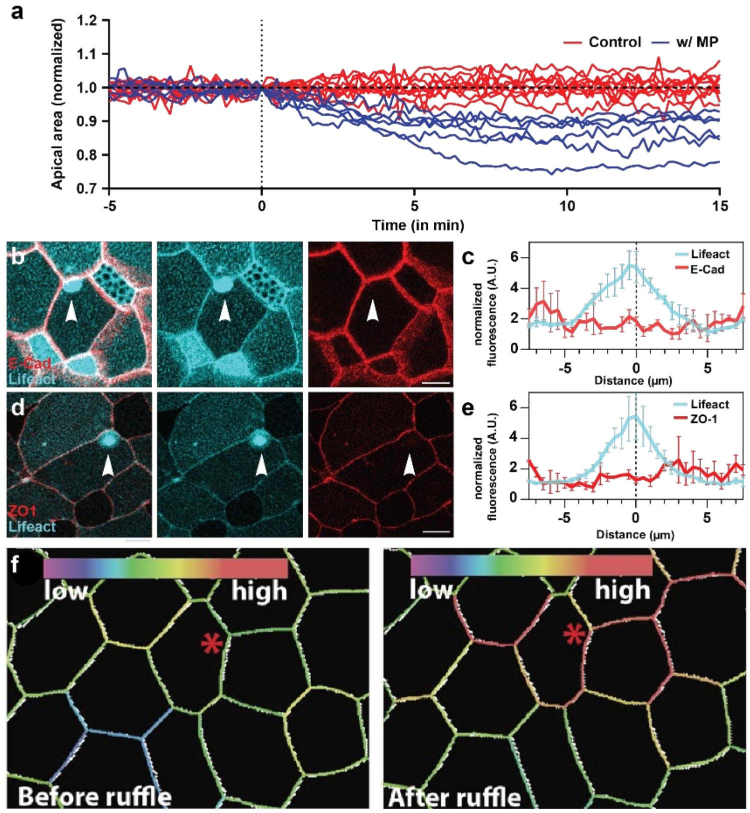

Supplementary Fig. 1. Macropinocytosis induces apical size reduction without junction protein internalization.
a Quantification of cell size change in cells with (blue) or without (red) a macropinocytotic event using cells synchronized to the onset of macropinocytosis and normalized to original cell size. All traces are from a single embryo, with each trace representing the apical size evolution of an individual cell (n = 10 cells without MP, 6 cells with MP). **b, d** Representative image of a tissue injected with (b) cadherin-FP (red) or (d) ZO1-FP (red) and Lifeact-FP (cyan) with a cell undergoing macropinocytosis. Scale bar 30 µm. **c, e** Quantification of the cadherin (c) or ZO1 (e) intensity associated with closing actin ruffles ((c) n = 3 exp. and 32 cells; (e) n = 2 exp., and 30 cells; point = mean; error bars = SD) **f** Inferred tension heatmaps using the CellFit program before (left) and after (right) a macropinocytotic event marked with a red asterisk.

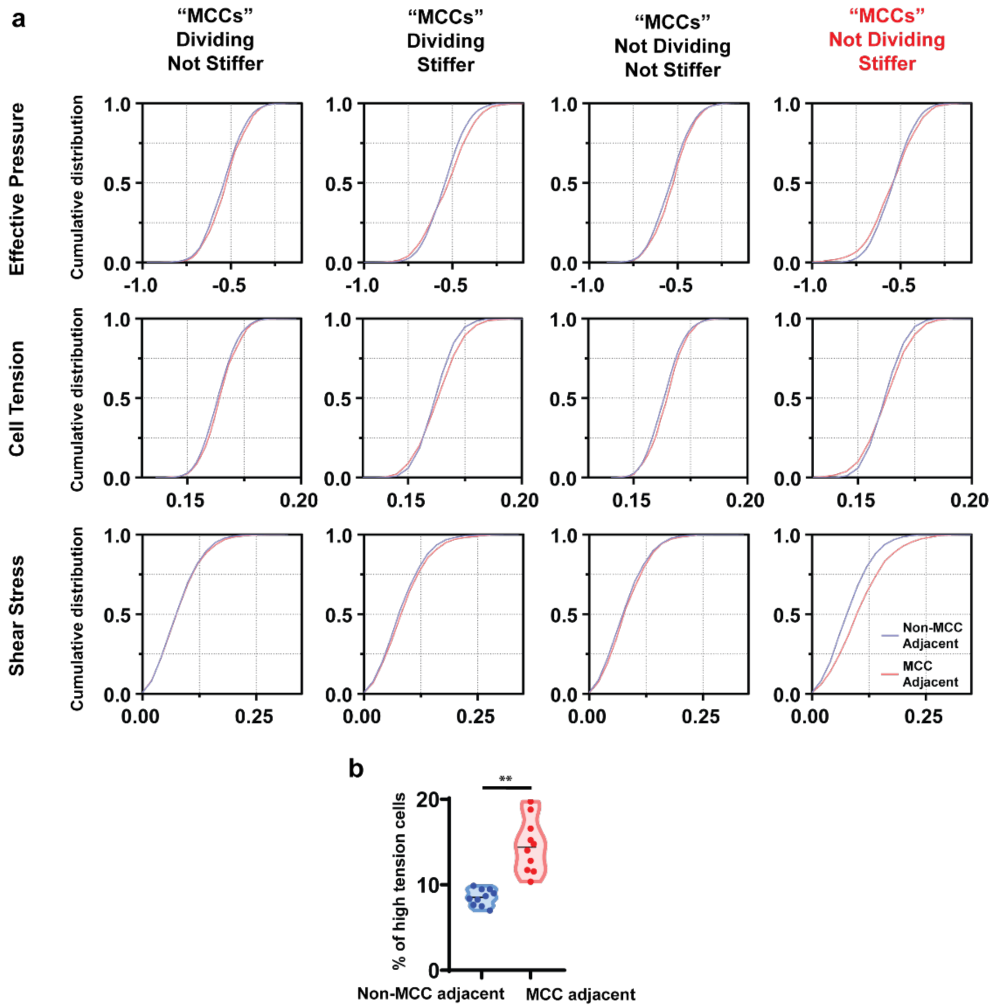

Supplementary Fig. 2. Simulations reveal mechanical heterogeneity around multiciliated cells
a Cumulative distribution of cell's effective pressure, tension or shear stress in simulated epithelium with multiciliated cells in name only (dividing and not stiffer), dividing and stiffer, not dividing and not stiffer, or not dividing and stiffer (n= 10 simulations per condition). **b** Percentage of non-MCC neighbors and MCC neighbors that are in the highest decile of tension (n = 10 simulations, two-sided paired t-test; p-value = 0.0011).

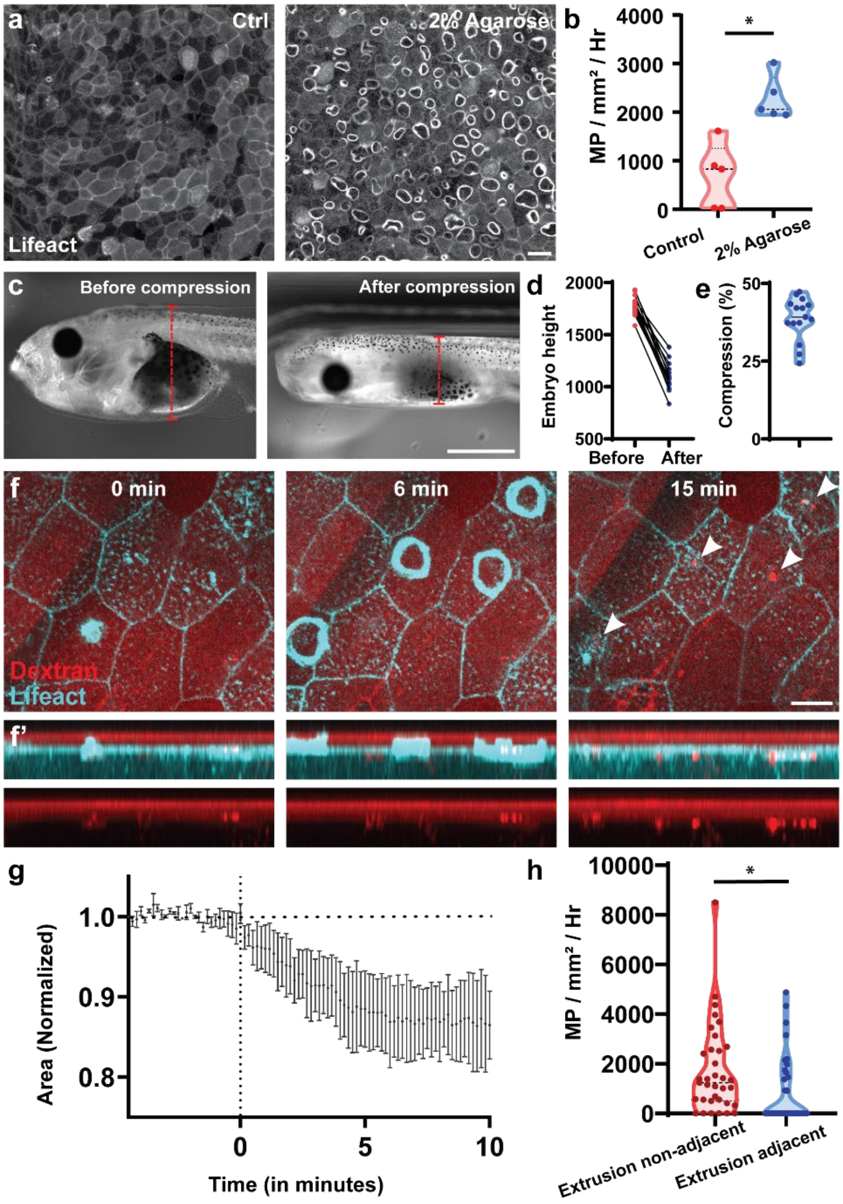

Supplementary Fig. 3. Macropinocytosis is rapidly induced by external compression.
a Representative image of an epithelium expressing Lifeact-FP before (left) and after (right) compression in 2% agarose. Scale bar 30 µm. **b** Quantification of macropinocytosis before and after embryo compression in 2% Agarose (n = 2 exp. and 5 embryos; two-sided paired t-test; p-value = 0.0143). **c** Images of the same embryo before and after compression. Scale bar 1 mm. **d** Quantification of embryo height before and after compression (n = 3 exp. and 15 embryos). **e** Quantification of embryo compression percentage (n = 3 exp. and 15 embryos). **f** Time lapse image of Lifeact-FP (cyan) showing macropinocytotic internalization of fluorescent dextran (red) from the media in embryo under compression. f' show is a z projection of the area. Scale bar 10 µm. **g** Quantification of cell size change in tissues where we find isolated cells having a macropinocytotic event upon compression (n = 3 exp. and 45 cells, error bars = SD). **h** Quantification of macropinocytosis level in compressed embryo in cell adjacent or non-adjacent to an extrusion event (n = 6 exp. and 30 extrusion events; two-sided paired t-test; p-value = 0.0163).
