# Supplementary material for: Apical size reduction by macropinocytosis alleviates tissue crowding
Source: Nat Commun. 2025 Jun 23;16:5338. doi: 10.1038/s41467-025-60724-2 (PMC12185762; doi:10.1038/s41467-025-60724-2)
Supplement: Supplementary file 2 — Description of Additional Supplementary Files [file 41467_2025_60724_MOESM2_ESM.pdf]

Description of Additional supplementary files

Supplementary Movie 1. Confocal microscopy movie of an embryo co-expressing membrane-FP (Red) in multiciliated cells and Lifeact-FP (Cyan) in all of the epidermis. Time step in 2 min.

Supplementary Movie 2. Confocal microscopy movie of an embryo expressing Lifeact-FP. Time step in 1 min.

Supplementary Movie 3. Confocal microscopy movie of an embryo expressing Lifeact-FP (Cyan) imaged in a media containing fluorescent Dextran (Red). Time step in 2 min.

Supplementary Movie 4. Confocal microscopy movie of an embryo co-expressing Lifeact-FP (Cyan) and active myosin II (SF9, Red). Time step in 2 min.

Supplementary Movie 5. Confocal microscopy movies of an explanted tissue expressing Lifeact-FP. Time step in 2 min.

Supplementary Movie 6. Confocal microscopy movies of an organoid expressing Lifeact-FP. Time step in 0.5 min.

Supplementary Movie 7. Confocal microscopy movie of cells having an MP event in embryos expressing Lifeact-FP (Cyan) imaged in a media containing fluorescent Dextran. Embryos are either treated with DMSO or GsMTx4, Sodium Deoxycholate, Mβ cyclodextrin or Yoda1. Time step in 2 min.

Supplementary Movie 8. A movie of a simulated epithelium with cells color-coded for effective pressure, cell tension or shear stress.

Supplementary Movie 9. Confocal microscopy movie of a compressed embryo co-expressing LifeactFP (Cyan) and E-Cadherin-FP (Red). Time step in 3 min.

Supplementary Movie 10. Confocal microscopy movie of a compressed embryo co-expressing LifeactFP (Cyan) and E-Cadherin-FP (Red) showing cell extrusion (asterisk). Time steps in 3 min.
